# Efficacy of rivaroxaban for pulmonary embolism

**DOI:** 10.1097/MD.0000000000015224

**Published:** 2019-04-19

**Authors:** Juan Jia, Shi-min Xue, Ning Xu

**Affiliations:** a Department of Respiratory Medicine; b Department of Critical Medicine, Yulin No. 2 Hospital, Yulin, Shaanxi, China.

**Keywords:** efficacy, pulmonary embolism, rivaroxaban, systematic review

## Abstract

**Background:**

Previous clinical trials have addressed that rivaroxaban is effective for the treatment of patients with pulmonary embolism (PE). This study will systematically assess its efficacy and safety for PE.

**Methods:**

We will carry out this study by searching the following electronic databases from inception to March 1, 2019 without language restrictions: Cochrane Library, EMBASE, PUBMED, Web of Science, Cumulative Index to Nursing and Allied Health Literature, Allied and Complementary Medicine Database, Chinese Biomedical Literature Database, and China National Knowledge Infrastructure. In addition, we will also search clinical trial registries, dissertations, and conference abstracts to avoid any missing potential studies. All randomized controlled trials of rivaroxaban for patients with PE will be fully considered. Two researchers will independently perform literature selection, data collection, and methodological quality assessment. If it is appropriate, outcome data will be pooled by using a fixed-effect model or random-effect model, and meta-analysis will be considered for operation.

**Results:**

All efficacy and safety of rivaroxaban for PE will be assessed through all primary and secondary outcomes. The primary outcomes are all-cause mortality and major bleeding. The secondary outcomes are recurrent venous thromboembolism, duration of hospital stay, quality of life, patient satisfaction, and adverse events.

**Conclusion:**

The findings of this study will summarize updated evidence on the efficacy and safety of rivaroxaban for patients with PE.

**Ethics and dissemination:**

It is not necessary to inquire ethical approval for this study, because it will not analyze any individual patient data. The results of this study will be published through peer-reviewed journals.

**Systematic review registration:**

PROSPERO CRD42019126095.

## 1 Introduction

Venous thromboembolism (VTE), which consists of deep vein thrombosis and pulmonary embolism (PE), is a very tricky condition at clinic.^[1–4]^ It is often associated with very high mortality and morbidity in patients with such condition.^[1,4]^ It has been reported that about 20% patients with VTE died before or one day after they diagnosed with VTE. Furthermore, about 11% of the VTE survivors still may die due to the variety of complications within 3 months.^[5,6]^ Of such condition, PE is a potential life-threatening disease, especially acute PE that requires urgent intervention. It is also the third leading reason of death in patients with cardiovascular diseases, following coronary heart attack, and stroke.^[7]^ It has been estimated that the incidence of PE is about 69 per 100,000 every year. Most importantly, its associated mortality risk is reported to more than 20% and 65% of deaths happening within 1 hour.^[8–10]^ Furthermore, such mortality risk may still persist with 5%, and lasts up to 12 months.^[11]^

Standard therapy for PE comprises of warfarin, a dose-adjusted vitamin K antagonist.^[12–14]^ It is an oral anticoagulant for the treatment of PE. However, its anticoagulation effect cannot work as immediate-acting parenteral agent until after 4–5 days treatment.^[15–17]^ Additionally, it also requires strict dietary, and regular monitor for anticoagulation effect to keep international normalized rate (INR) within the range of 2.0–3.0.^[18]^

Rivaroxaban is a specific developed oral anticoagulant with rapid onset of action.^[19]^ It is also a direct Factor Xa inhibitor, which does not need for routine INR monitoring.^[19,20]^ A numerous clinical trials have reported that rivaroxaban is effective for patients with PE.^[21,22]^ Furthermore, although a systematic review has addressed to assess the benefits of rivaroxaban for PE involving observational studies only, and search date up to November 2016,^[23]^ lots of high quality randomized controlled trials (RCTs) were published after that study.^[24–38]^ Thus, this updated study will systematically evaluate the efficacy and safety of rivaroxaban for PE, and will provide its latest updated evidence.

## 2 Methods

### 2.1 Study registration

This study has been registered on PROSPERO (CRD42019126095). The systematic review protocol will be reported in accordance with the Preferred Reporting Items for Systematic Reviews and Meta-Analysis Protocol statement guidelines.^[39]^

### 2.2 Eligibility criteria

#### 2.2.1 Types of studies

This study will only consider RCTs. Any other studies will be not considered, such as nonclinical studies, noncontrolled trials, crossover studies, non-RCTs, and quasi-RCTs.

#### 2.2.2 Types of participants

Participants with a diagnosis of PE will be included regardless of race, gender, sex, and their economic status.

#### 2.2.3 Types of interventions

##### 2.2.3.1 Experimental interventions

We will include any forms of rivaroxaban monotherapy. Any combination of rivaroxaban and other treatments will not be considered.

##### 2.2.3.2 Control interventions

Apart from rivaroxaban, therapies in control group can be any treatments.

#### 2.2.4 Type of outcome measurements

##### 2.2.4.1 Primary outcomes

All-cause mortality;

Major bleeding (as assessed by simplified Pulmonary Embolism Severity Index score, or other instruments).

##### 2.2.4.2 Secondary outcomes

Recurrent venous thromboembolism;

Duration of hospital stay;

Quality of life (as evaluated by Global Quality of Life Scale or other tools);

Patient satisfaction (as measured by generic Treatment Satisfaction Questionnaire for Medication version II or other scales);

Adverse events (any expected and unexpected adverse events).

### 2.3 Search strategy

Cochrane Library, EMBASE, PUBMED, Web of Science, Cumulative Index to Nursing and Allied Health Literature, Allied and Complementary Medicine Database, Chinese Biomedical Literature Database, and China National Knowledge Infrastructure will be searched from inceptions to March 1, 2019 without language restrictions. Additionally, clinical trial registries, dissertations, and conference abstracts will also be searched. The detailed search strategy of Cochrane Library is demonstrated in Table 1. Similar strategies will be made and utilized to any other electronic databases.

**Table 1:**
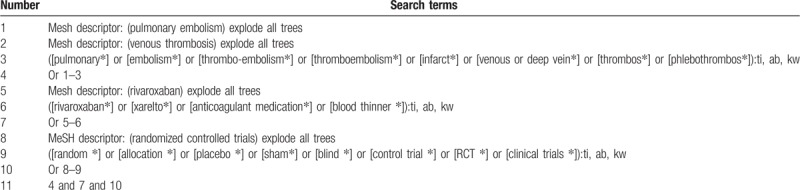
Search strategy used in Cochrane Library database.

### 2.4 Study selection

Two researchers will perform all study selection independently. Any conflict regarding the study selection between two researchers will be solved by a third researcher through discussion. At the first stage, they will review the titles and abstracts of all records to identify any potential studies based on the predefined eligibility criteria. At the second stage, full-texts of all potential relevant studies will be read for further judgments. The whole study selection procedure will abide to the PRISMA guidelines, and will be showed in PRISMA flowchart.

### 2.5 Data extraction and management

A standard data extraction form will be created before the data collection. Two researchers will independently collect all the important information and extract data from each eligible study. Any conflicts between two researchers will be settled down by consulting a third researcher. The extracted information includes: general characteristics of eligible studies (e.g., first author name, published year, country, sample size, eligibility criteria, and so on), details of patients (e.g., sex, age, and so on), details of treatments (e.g., drug, dosage, frequency, route, and so on), and outcomes (e.g., primary, secondary, and other outcome measurements).

### 2.6 Missing data

Any missing data will be required by contacting primary authors with emails. If we cannot receive those data, we will only analyze available data, and will discuss it.

### 2.7 Methodological quality assessment

Cochrane risk of bias tool will be used to evaluate the methodological quality for each eligible trial. It will be divided into 7 aspects, and each aspect will be classified as low risk, unclear, or high risk. Two independent researchers will perform the whole process of methodological quality assessment. If there are disagreements, a third researcher will help to resolved them by discussion.

### 2.8 Strategy for data synthesis

#### 2.8.1 Measurement of treatment effect

As for dichotomous values, we will record them as risk ratio with 95% confidence intervals (CIs). As for continuous values, we will record them as mean difference or standardized mean difference with 95% CIs.

#### 2.8.2 Data synthesis

We will judge heterogeneity by using *I*^*2*^ test. If *I*^*2*^ is less than 50%, low heterogeneity is regarded. Then, we will use a fixed-effect model to synthesize the data. Meanwhile, meta-analysis will be carried out by using RevMan version 5.3 software. If *I*^*2*^ is more than 50%, high heterogeneity is considered, and subgroup analysis will be conducted. If there is low heterogeneity after subgroup analysis, we will pool the data by using a random-effect model, and we will also carry out meta-analysis. If there is still high heterogeneity after subgroup analysis, we will not synthesize the data, and only a narrative summary will be reported.

#### 2.8.3 Subgroup analysis

Subgroup analysis will be carried out based on the different treatments, controls, and outcomes.

#### 2.8.4 Sensitivity analysis

Sensitivity analysis will also be performed to check the robustness of pooled results by eliminating the impact of low-quality studies.

#### 2.8.5 Reporting bias

The funnel plot and Egger's test will be performed to evaluate the reporting bias if at least 10 trials are included.

## 3 Discussion

Although a previous relevant study has been published in 2017,^[23]^ it only included observational studies up to the November 2016 with quite low level of evidence, and lots of high-quality RCTs have been published after that study.^[24–38]^ This updated study protocol will only include higher level evidence of RCTs, and will systematically assess the efficacy and safety of rivaroxaban for patients with PE. Its results will summarize the latest updated evidence on assessing the efficacy and safety of rivaroxaban for PE. It may also present solid data and robust evidence either to the clinical practice, and future studies, or to the health policy makers.

## Author contributions

**Conceptualization:** Juan Jia, Ning Xu.

**Data curation:** Juan Jia, Shi-min Xue, Ning Xu.

**Formal analysis:** Juan Jia, Shi-min Xue.

**Funding acquisition:** Ning Xu.

**Investigation:** Ning Xu.

**Methodology:** Juan Jia, Shi-min Xue.

**Project administration:** Ning Xu.

**Resources:** Juan Jia, Shi-min Xue.

**Software:** Shi-min Xue.

**Supervision:** Ning Xu.

**Validation:** Juan Jia, Shi-min Xue, Ning Xu.

**Visualization:** Juan Jia, Shi-min Xue, Ning Xu.

**Writing – original draft:** Juan Jia, Shi-min Xue, Ning Xu.

**Writing – review & editing:** Juan Jia, Shi-min Xue, Ning Xu.
